# The relation between preterm birth and self-reported spinal pain in pre-adolescence—a study of 47,063 subjects from the Danish National Birth Cohort

**DOI:** 10.1007/s00431-023-05264-x

**Published:** 2023-10-20

**Authors:** Klara Kaltoft, Jane Lykke Nielsen, Anne-Marie Nybo Andersen, Anne Cathrine Falch-Joergensen

**Affiliations:** Section of Epidemiology, Department of Public Health, Faculty of Health and Medical Science, University of Copenhagen, Oster Farimagsgade 5, bd. 24, DK-1014 Copenhagen K, Denmark

**Keywords:** Back pain, Neck pain, Preterm, Children, Adolescents

## Abstract

Repeated exposure to pain and stress in early life may cause alterations in pain sensitivity later in life. Children born preterm are often exposed to painful invasive procedures. This study aimed to explore the relationship between being born preterm and self-report of spinal pain in pre-adolescence. This prospective study was based on the Danish National Birth Cohort and consisted of 47,063 11–14-year-olds. Data from the Danish National Birth Cohort were linked with national registers through Statistics Denmark. Analyses were performed as multiple logistic regression models estimating odds ratios and 95% confidence intervals. Spinal pain (neck, mid back, and/or low back pain) was assessed using a subdivision of the Young Spine Questionnaire. Severe spinal pain was defined as having pain often or once in a while with an intensity of four to six on the Revised Faces Pain Scale. Inverse probability weighting was used to account for potential selection bias. Girls born very preterm (< 34 full weeks of gestation) were less likely to report spinal pain (OR: 0.60; 95% CI: 0.40–0.93) compared with those term-born. The associations were weaker when examining moderate to severe spinal pain and when examining the three spinal regions separately. None of these was statistically significant.

*Conclusion*: We found no associations for boys. In conclusion, this study indicates that girls born very preterm are seemingly less likely to have severe spinal pain in pre-adolescence than girls born at term.

**Table:** 

**What is Known:** *• Spinal pain is one of the largest disease burdens globally, and the evidence regarding the etiology of spinal pain in children and adolescents is limited.* *• Repeated exposure to pain and stress in early life (i.e., being preterm) may cause alterations in pain sensitivity later in life.*	
**What is New:** *• Girls born very preterm (< 34 full weeks of gestation) seem less likely to report severe spinal pain in pre-adolescence compared with girls born at full term.* *• There is no association between gestational age and later experience of spinal pain in pre-adolescent boys.*

**Supplementary Information:**

The online version contains supplementary material available at 10.1007/s00431-023-05264-x.

## Introduction

Spinal pain (i.e., low back, mid back, and/or neck pain) is one of the largest disease burdens globally [1], with back pain being the leading cause of years lived with disability [2]. Spinal pain in adults is well documented [3]. Even though there have been studies regarding spinal pain in children, the evidence remains limited primarily due to methodological limitations and the lack of comparability across these studies [4]. As a result, the reported prevalence of back pain in children and adolescents varies widely, ranging from 7 to 72% [4]. The wide variability in prevalence can be attributed to several factors, including disparities in study populations, sample size, study period, and methodological approaches which complicate compatibility between studies [4]. The findings regarding several risk factors for spinal pain in children are mixed [4], but studies have shown that risk factors for spinal pain include biological, social, and psychological factors [4, 5]. It is further documented that children who experience low back pain often also experience low back pain later in life [6], and that recurrent episodes of back pain may not be a serious of unrelated episodes, but rather a long-term condition [5].

Experience of early life pain is associated with spinal pain among pre-adolescents [7]. Preterm born children often undergo several invasive procedures. These procedures often occur during a period where the brain is under great development and particularly vulnerable for alterations [8]. Repeated exposure to pain and stress in the first years of life can alter the neurological substrate associated with pain perception, which consequently can affect somatosensory processing of pain and change neurobehavioral responses to pain [7, 9–12]. Building on these findings, the present study was based on the hypothesis that pre-adolescents born preterm may be more sensitive to pain due to early-life pain experiences, resulting in distinct pain perception compared to their term-born peers. Furthermore, preterm birth is known to affect both short- and long-term health outcomes [13–15], and children born preterm are at risk of developing late effects of a physical, mental, motor, social, and cognitive nature [13–15], which have likewise been associated with higher risk of spinal pain [4, 5]. However, despite this, findings from an epidemiological cohort study have indicated that adults born preterm had a lower risk of reporting musculoskeletal pain, including spinal pain [16].

The overall aim of this study was to explore the relationship between being born preterm and spinal pain in pre-adolescence (11–14 years old, with the majority being 11-year-olds) taking advantage of the large birth cohort The Danish National Birth Cohort (DNBC) as is one of the few databases including information on spinal pain. Specifically, we aimed to examine the association between preterm birth and severe spinal pain among 11–14-year-old boys and girls, respectively, and further whether the association differed according to severity and localization of pain.

## Material and methods

### Study population

**Fig. 1:**
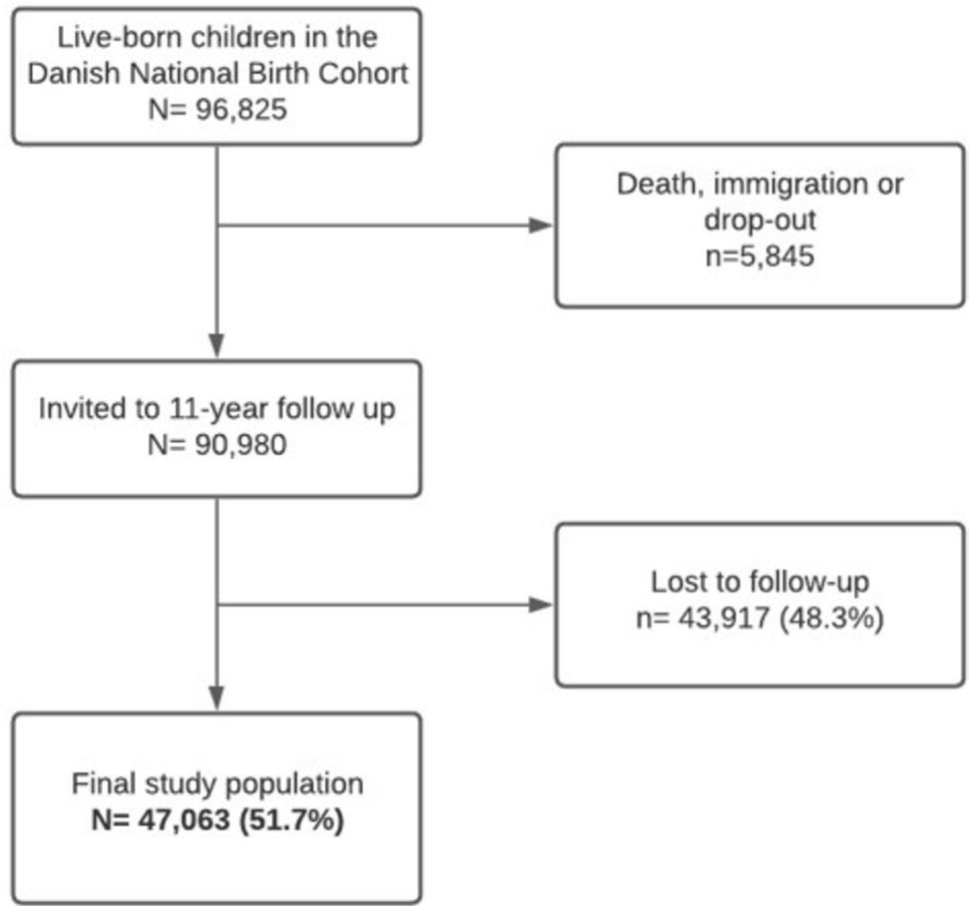
Flow chart of study population

DNBC is a cohort consisting of approximately 100,000 children born from 1996 to 2003 and their mothers, followed by several data collections from early pregnancy through the children’s life [17]. When the children turned 11 years old, they got invited to participate in the 11-year follow-up (DNBC-11), which included questions about spinal pain [17, 18]. The source population consisted of all pre-adolescents included in DNBC. In DNBC-11, a total of 47,063 pre-adolescents contributed with full information on all explanatory measures and spinal pain (Fig. 1); thus, all missing data were excluded. Data from DNBC were linked with nationwide register data on Statistics Denmark through the unique personal identification number assigned to all persons with a permanent residency in Denmark. All data were stored and processed in the secure remote server environment at Statistics Denmark.

### Preterm birth

Gestational age at birth was obtained from the Medical Birth Register containing information on all births in Denmark [19]. Gestational age was operationalized into full weeks and further into the following three groups: term (≥ 37 full weeks of gestation), moderate preterm (34–36 full weeks of gestation), and very preterm (< 34 full weeks of gestation).

### Spinal pain in pre-adolescence

Spinal pain was assessed in DNBC-11 in which a subdivision of the Young Spine Questionnaire (YSQ) was included. YSQ is a validated questionnaire used to measure the intensity and prevalence of pain in the neck, mid back, and lower back [20]. The original questionnaire consists of 19 questions, of which nine were included in DNBC-11. Frequency was measured by the following options: (1) often, (2) once in a while, (3) once or twice, and (4) never. Intensity was measured by the Revised Faces Pain Scale and ranged from 1 = no pain to 6 = very much pain [20]. There is no verified classification of spinal pain among children but in this study, we used an overall combined measure, which was divided into no pain, moderate pain, or severe pain in the low back, mid back, or neck. This measure was directly adopted from previous work based on the same population [7, 18, 21]. For further detailed information regarding the operationalization of the variable, see the previous study by Joergensen et al. [18]. Thus, severe pain was defined as having pain often or once in a while with an intensity of four or more on the Revised Faces Pain Scale [18, 22]. If the child reported pain in more than one region, the region in which the child reported the most severe pain was included in the outcome. In the main analysis, the outcome was divided into two groups: no pain (including moderate pain) versus severe spinal pain. Secondly, we operationalized a variable in which having experienced spinal pain covered both moderate and severe spinal pain. This variable included having experienced pain once or twice or once in a while or often with an intensity of 3 or more or having pain often with an intensity of 1 or more [18]. The three spinal regions were also investigated separately.

### Covariates

With the use of a causal diagram, potential confounders were selected a priori [23] (Supplementary File 1). The selected confounders were parity, maternal age at childbirth, major congenital anomalies, and parental educational level. Information on sex was obtained from DNBC. Age corresponded to the child’s age when responding to DNBC-11. Parity and maternal age at birth were obtained from the Danish Medical Birth Register. Parity was divided into nulliparous or parous. Maternal age at birth was divided into the following groups: < 25, 25–29, 30–34, and > 34 years old. Major congenital anomalies originated from the Medical Birth Register and were defined according to the EuroCAT guidelines [24]. Parental educational level was obtained from The Danish Population’s Education Register [25] and described the highest ongoing or completed educational level of the parents. It was categorized into low (0–2 ISCED), medium (3–4 ISCED), and high (5–8 ISCED) educational level according to the International Standard Classification of Education (ISCED) 2011 [26].

### Statistical methods

All statistical analyses were made using STATA V.16.1. Initially, we made descriptive statistics between the exposure of interest and the selected covariables. Categorical variables were reported as proportions and tested for heterogeneity using a chi-squared test. Applying multiple logistic regression models, we examined crude and adjusted associations between preterm birth and severe spinal pain in pre-adolescents aged 11 to 14 years. We estimated odds ratios (OR) and an *α*-level of 0.05 in the statistical testing was used, corresponding to a 95% confidence interval (95% CI). In all analyses, no spinal pain was considered as the reference outcome. We tested the interaction between preterm birth and sex (*p* = 0.160) and based on this, we could not definitively reject a possible interaction with sex, and therefore we chose to divide the analyses by sex. The analyses further accounted for the dependency between siblings in the study sample including a robust standard error estimator [27]. Subsequently, we analyzed the association by using the secondary outcomes: “moderate to severe” as well as the spinal regions separately. We performed a loss-to-follow-up analysis to investigate potential selection bias in our analysis population. To this, we used chi-squared tests of heterogeneity to compare study participants with individuals lost to follow-up (Supplementary File 2). In a sensitivity analysis, we further accounted for sample selection (into the cohort and from attrition) by applying inverse probability weighting (IPW) using a reference population consisting of all children born in Denmark between 1996 and 2003 [28, 29]. These analyses were conducted in line with previous spinal pain studies performed in the same study population [7, 18, 30].

### Approval of the study

Approval of the study was obtained from the Danish Data Protection Agency through the joint notification of The Faculty of Health and Medical Sciences at The University of Copenhagen (SUND-2017–09) and the DNBC Steering Committee (2017–23). All data were stored and processed at Statistics Denmark and no personally identifiable data were accessible.

## Results

Table 1 shows characteristics of pre-adolescents born at term, moderate preterm, and very preterm. In this study population, almost 6% of boys and 5% of girls were born moderate or very preterm. Children born preterm were more often the firstborn child, had mothers below 25 years or over 35 years, and had parents of lower educational level compared to children born to term (Table 1). Boys born very preterm were more likely to have a major congenital anomaly compared to boys born moderate preterm or at term.

**Table 1:** Characteristics of the 47,063 pre-adolescents included in the study population stratified by child’s sex (The Danish National Birth Cohort, 1996–2003)

**Characteristics**	**Term** **%**	**Moderate preterm** **%**	**Very preterm** **%**	Total %
**Boys**^**a**^ ***N*** **(%)**	21,163 (94)	972 (4.3)	304 (1.4)	22,439 (100)
**Age at DNBC-11**				
11 years	82	84	85	82
12 years	15	13	12	15
13 + years	2.6	2.6	3.6	2.7
**Parity**				
Nulliparous	48	61	68	48
Parous	53	39	32	52
**Maternal age at birth**				
< 25 years	5.4	5.8	7.2	5.4
25–29 years	35	33	30	35
30–34 years	41	39	41	41
≥ 35 years	19	23	22	19
**Major congenital anomalies**				
Yes	3.9	6.1	10.2	4.1
No	96	94	90	96
**Parental education**				
High	65	59	61	64
Medium	33	39	35	33
Low	2.4	2.7	4.6	2.4
**Girls**^**a**^ ***N*** **(%)**	23,383 (95.0)	935 (3.9)	276 (1.1)	24,624 (100)
**Age at DNBC-11**				
11 years	82	82	84	82
12 years	16	15	14	16
13 + years	2.5	2.7	2.2	2.5
**Parity**				
Nulliparous	47	60	68	48
Parous	53	40	32	52
**Maternal age at birth**				
< 25 years	5.6	5.9	6.9	5.6
25–29 years	35	36	30	35
30–34 years	41	38	38	41
≥ 35 years	19	20	25	19
**Major congenital anomalies**				
Yes	3.1	4.5	3.6	3.2
No	97	96	96	97
**Parental education**				
High	63	61	54	63
Medium	35	36	41	35
Low	2.6	2.9	4.4	2.6

We used chi-squared tests of heterogeneity to analyze variables. Chi-squared tests were statistically significant for all variables except for major congenital anomalies and maternal age at birth among girls

^a^Data are shown in row percentages

The prevalence of spinal pain among boys was 9.8% and 14% among girls (data not shown), where neck pain was the prevailing spinal pain site for both boys and girls (Supplementary file 3). We observed no association between moderate nor very preterm birth and severe spinal pain in boys (OR_very preterm_: 0.99, 95% CI: 0.66–1.48) (Table 2). For girls, however, we found that those born very preterm were less likely to report severe spinal pain in pre-adolescence compared to those born to term (OR = 0.60, 95% CI: 0.40–0.93). The association with moderate preterm appeared weaker and was not statistically significant.

**Table 2:** Odds ratio (OR) of severe spinal pain among the 47,063 pre-adolescents in the study of preterm birth and spinal pain in pre-adolescence stratified by child’s sex (The Danish National Birth Cohort, 1996–2003)

**Characteristics**	**Spinal pain**		
	Total (%)	Crude OR [95% CI]	Adjusted^a^ OR [95% CI]
**Boys**			
Term	2074 (94.3)	Ref	Ref
Moderate preterm	96 (4.4)	1.01 [0.81–1.25]	1.02 [0.82–1.27]
Very preterm	29 (1.3)	0.97 [0.64–1.45]	0.99 [0.66–1.48]
**Girls**			
Term	3305 (95.8)	Ref	Ref
Moderate preterm	119 (3.5)	0.85 [0.70–1.04]	0.86 [0.71–1.05]
Very preterm	25 (0.7)	0.61 [0.40–0.92]	0.60 [0.40–0.93]

^a^Adjusted for parity, major congenital anomalies, maternal age at birth, and parental education

In addition, we observed no association for neither boys nor girls when the outcome measure consisted of “moderate to severe” spinal pain (Table 3), or when investigating the spinal regions separately (Table 4). However, there was an indication that the observed association between very preterm birth and severe spinal pain seemed mainly driven by neck pain (Table 4).

**Table 3:** Odds ratio (OR) of moderate or severe spinal pain among the 47,063 pre-adolescents in the study of preterm birth and spinal pain in pre-adolescence stratified by child’s sex (The Danish National Birth Cohort, 1996–2003)

**Characteristics**	**Spinal pain**	
	Total (%)	Adjusted^a^ OR [95% CI]
**Boys**		
Term	8177 (94.1)	Ref
Moderate preterm	397 (4.6)	1.12 [0.98–1.28]
Very preterm	117 (1.4)	1.02 [0.80–1.30]
**Girls**		
Term	10,422 (95.7)	Ref
Moderate preterm	411 (3.8)	0.93 [0.82–1.06]
Very preterm	110 (1.0)	0.83 [0.65–1.06]

^a^Adjusted for parity, major congenital anomalies, maternal age at birth, and parental education

**Table 4:** Odds ratio (OR) of severe pain in the neck, mid back, and low back, respectively, among the 47,063 pre-adolescents in the study of preterm birth and spinal pain in pre-adolescence stratified by child’s sex (The Danish National Birth Cohort, 1996–2003)

**Characteristics**	**Neck pain**		**Mid back pain**		**Low back pain**	
	Total (%)	Adjusted^a^ OR [95% CI]	Total (%)	Adjusted^a^ OR [95% CI]	Total (%)	Adjusted^a^ OR [95% CI]
**Boys**						
Term	1353 (94.2)	Ref	707 (94.1)	Ref	537 (93.6)	Ref
Moderate preterm	63 (4.4)	1.02 [0.78–1.33]	34 (4.5)	1.06 [0.74–1.50]	31 (5.4)	1.29 [0.89–1.86]
Very preterm	20 (1.4)	1.03 [0.64–1.66]	10 (1.3)	1.00 [0.53–1.89]	6 (1.1)	0.80 [0.36–1.79]
**Girls**						
Term	2002 (96.2)	Ref	1188 (95.2)	Ref	1116 (95.4)	Ref
Moderate preterm	65 (3.12)	0.77 [0.60–1.00]	50 (4.0)	1.04 [0.79–1.39]	45 (3.9)	0.98 [0.72–1.34]
Very preterm	15 (0.7)	0.61 [0.35–1.06]	10 (0.8)	0.72 [0.38–1.35]	9 (0.8)	0.67 [0.35–1.31]

^a^Adjusted for parity, major congenital anomalies, maternal age at birth, and parental education

Information on the pre-adolescents lost to follow-up is shown in supplementary file 2. Pre-adolescents who were lost to follow-up accounted for 48% of the source population. The chi-squared test showed that the study population differed from the population lost to follow-up. Those lost to follow-up were more often boys, born preterm, from a nulliparous mother, and their mothers were more often < 25 years old at birth. Furthermore, their parents were of lower educational background. Even though the results of the loss-to-follow-up analysis revealed signs of selection, the IPW analyses in which we accounted for potential selection into the cohort and from attrition showed no essential changes to the estimates (data not shown).

## Discussion

In this study of more than 45,000 individuals aged 11 to 14, we examined the association between preterm birth and spinal pain in pre-adolescence. Contrary to our expectations, this study suggested that girls born very preterm were less likely to report spinal pain in pre-adolescence than girls born at full term. There was no association for boys or for the spinal regions examined separately. When the outcome of spinal pain included both moderate and severe spinal pain, we found no association among boys nor girls.

This study was based on the hypothesis that pre-adolescents born preterm may be pain sensitized in early life due to several exposures to painful experiences and that their experience of pain, therefore, may differ from pre-adolescents born at term. Several studies suggest that pain sensitivity may be affected in early life and that this may affect how pain is experienced later in life [7, 9, 10, 31, 32]. Based on this knowledge, we expected children born preterm to experience spinal pain to a greater extent compared to children born at term; however, we observed the contrary relationship. We identified one study indicating that children who had several pain experiences in early life were more resilient to pain later in life [9]. However, the differences in findings from these studies may be explained by differences in study designs, study populations varying between 26 and 29,861 subjects, and differences in measurements assessing early-life pain and pain sensitivity [7, 9, 10, 31, 32].

Epidemiological studies have shown an association between puberty stage and back pain in teenagers [33, 34]. Puberty-related changes in hormone levels and subsequent physical, mental, and emotional changes [35] and possibly also stress [36] usually begin around the age of 10–11 in girls and a couple of years later in boys. Studies have shown that girls born preterm have a later onset of puberty than girls born at term [37, 38]. This together with the fact that both stress and poor well-being have been associated with spinal pain [21] could partly explain our findings that preterm girls have less spinal pain [39, 40]. Furthermore, a study examining spinal pain trajectories in children aged 6 to 17 years identified five trajectories with more advanced pubertal development being associated with both rare and moderate increasing pain trajectories [41]. Given that girls born preterm may have a later onset of puberty [37, 38], it is possible that they are more likely to follow a lower pain trajectory compared to their term-born peers. Hence, pubertal timing may be on the causal pathway between gestational age and spinal pain which is why we did not adjust for pubertal stage in this study.

Studies have found a correlation between height and respectively spinal pain and low back pain, where tall people reported pain to a greater extent [33, 42]. Children born preterm can have impaired height growth [43] which could be a part of the explanation of the reduced risk of reporting severe spinal pain among girls born preterm. This could potentially also be relevant for boys later in life, since they have a later growth spurt than girls. A study has found an increased odds for preterm born children to have impaired fine motor skills compared to a matched comparison group [44] and another study has linked poor motor skills at age 7 to neck and mid back pain at age 11 [45]. This may lead to preterm born children reporting spinal pain to a greater extent than children born to term. This does, however, not explain our results.

In addition to our hypothesis of pain sensitization in pre-adolescents born preterm, we also had an assumption that preterm birth may result in parental modeling of pain due to the parents handling and raising the child in a special way, because of a difficult beginning of life. A study describes that biological, psychological, and sociocultural factors are mechanisms underlying the experience of pain [31]. The child’s pain experience can be affected by their parent’s behavior, such that unnecessary worrying and protection can be a negative factor in children’s perception of pain [31, 46].

Being born preterm can lead to various long-term side effects [13–15]. Several of these consequences can be experienced worse than spinal pain and therefore may spinal pain not be in focus. This is supported by a study describing that children who had been admitted to a neonatal care unit reported less pain compared with children born term who had not been admitted to the neonatal care unit [9]. The children perceived pain to the same degree, but children admitted to a neonatal care unit avoided to report their pain, which is why they can be considered more robust or more likely to avoid reporting their pain [9].

### Strength and limitations

A strength of this study was the large study population, which provided a high degree of statistical power. Despite the large study population, only a small number of the children were born preterm which may not provide enough statistical power to assess the association between preterm birth and spinal pain. Additionally, it is worth noticing that the small numbers in some of the exposure categories and the many performed tests might have led to chance findings and that some of the statistically significant findings could be type 1 errors [47]. A further strength was the prospective study design ensuring temporality and further minimal risk of recall bias, since exposure data were based on register data and outcome data were collected as point prevalence in DNBC-11. Spinal pain was self-reported and pain experience is by default subjective, which may affect the accuracy of the variable [48]. Self-report has, however, been demonstrated as the best option to measure pain in children [49]. Additionally, data on spinal pain originated from YSQ, included in DNBC-11, which is validated to be used to measure the neck, mid back, and low back pain in 9–11-year-olds [20]. Finally, access to Statistic Denmark registers allowing the application of high-quality Danish population registers to obtain information on health and social conditions to adjust for potential confounders strengthened the validity of the study [19, 24, 25]. However, we can never be sure that we completely cover all variables that may confound the relationship.

As always there is selection into the cohort and from attrition, also demonstrated in the loss-to-follow-up analysis, which may have affected the study findings. However, the fact that children born preterm in this study figure in the same level as in the total Danish population is a strength [50]. In addition, a study investigating whether low participation in cohort studies is inducing bias suggested that non-participation did not affect the risk estimates in the DNBC cohort [51]. Finally, to account for potential selection bias, we performed IPW analyses, and as in previous studies in the same population, IPW did not make any essential changes to the estimates; thus, we believe that selection bias had no or a minimum influence on our results.

### Conclusion

In contrast to our hypothesis, this study indicates that girls born very preterm were less likely to have severe spinal pain in pre-adolescence than girls born to term, whereas there is seemingly no association between gestational age at birth and spinal pain in boys aged 11–14 years.

### Supplementary Information

Below is the link to the electronic supplementary material.

Supplementary file1 (DOCX 93 KB)

## Data Availability

Access to data requires permission from the Danish Data Protection Agency and the DNBC Steering Committee. Please see https://www.dnbc.dk/access-to-dnbc-data for further information.

## Abbreviations

DNBC: The Danish National Birth Cohort
DNBC-11: The 11-year follow-up of the Danish National Birth Cohort
ISCED: The International Standard Classification of Education
OR: Odds ratio
YSQ: Young Spine Questionnaire
95% CI: 95% Confidence intervals

## Declarations

### Content to participate

Parents of the participants (< 18 years) signed an informed consent before participating in the study. No personal identifiable information was accessible.
