# Supplementary material for: The relation between preterm birth and self-reported spinal pain in pre-adolescence—a study of 47,063 subjects from the Danish National Birth Cohort
Source: Eur J Pediatr. 2023 Oct 20;183(1):203–11. doi: 10.1007/s00431-023-05264-x (PMC10857978; doi:10.1007/s00431-023-05264-x)
Supplement: Supplementary file 1 — Supplementary file1 (DOCX 93 KB) [file 431_2023_5264_MOESM1_ESM.docx]

**Supplementary File 1:** Causal diagram *of a priori selected potential confounders, which is an independent cause of the outcome and associated with the exposure.*


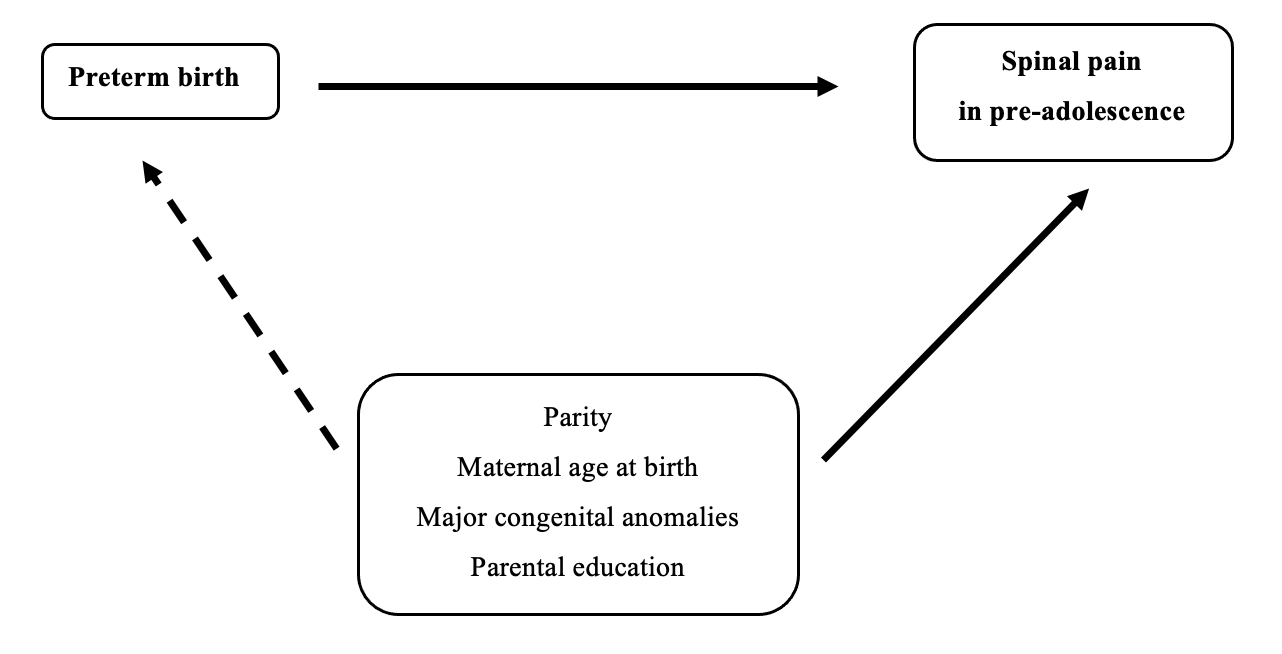


Supplementary File 2

| **Loss to follow-up *(The Danish National Birth Cohort, 1996-2003)*** | | |  |
| --- | --- | --- | --- |
| Characteristics | **Study population**  47,063 (51.7) | **Lost to follow-up^a^**  43,917 (48.3) | |
| Child’s sex |  |  | |
| Boys | 22,439 (47.7) | 24,098 (54.9) | |
| Girls | 24,624 (52.2) | 19,819 (45.1) | |
|  |  |  | |
| Gestational age |  |  | |
| Term | 44,546 (94.7) | 41,101 (93.6) | |
| Moderate preterm | 1,937 (4.1) | 2,059 (4.7) | |
| Very preterm | 580 (1.2) | 757 (1.7) | |
|  |  |  | |
| Parity |  |  | |
| Nulliparous | 22,607 (48.0) | 20,091 (45.8) | |
| Parous | 24,456 (52.0) | 23,766 (54.2) | |
| Missing | 0 | 60 | |
|  |  |  | |
| Maternal age at birth |  |  | |
| < 25 years | 2,586 (5.5) | 4,269 (9.7) | |
| 25-29 years | 16,371 (34.8) | 15,865 (36.1) | |
| 30-34 years   ≥ 35 years | 19,123 (40.6)  8,983 (19.1) | 16,590 (37.8)  7,193 (16.4) | |
|  |  |  | |
| Parental education |  |  | |
| High | 29,847 (63.4) | 20,892 (47.7) | |
| Medium | 16,028 (34.1) | 20,253 (46.2) | |
| Low | 1,188 (2.5) | 2,697 (6.2) | |
| Missing | 0 | 75 | |
|  |  |  | |
| Major congenital anomalies |  |  | |
| No | 45,371 (96.4) | 42,084 (95.8) | |
| Yes | 1,692 (3.6) | 1,833 (4.2) | |
|  |  |  | |
| a We used chi-squared tests of heterogeneity to compare study participants with individuals lost to follow-up. Chi-squared tests were statistically significant for all variables | | |  |

Supplementary File 3

**Prevalence of all types of spinal pain measured**

| **Characteristics** |  | **Neck pain** |  | **Mid back pain** |  | **Low back pain** |
| --- | --- | --- | --- | --- | --- | --- |
|  | Total (%) | Prevalence | Total (%) | Prevalence | Total (%) | Prevalence |
| **Boys (n=22,439)** |  |  |  |  |  |  |
| Term | 1,353 (94.2) | **6 %** | 707 (94.1) | 3.2% | 537 (93.6) | 2.4 % |
| Moderate preterm | 63 (4.4) | 0.3 % | 34 (4.5) | 0.2 % | 31 (5.4) | 0.1 % |
| Very preterm | 20 (1.4) | 0.1 % | 10 (1.3) | >0.1 % | 6 (1.1) | >0.1 % |
| **Girls (n=24,624)** |  |  |  |  |  |  |
| Term | 2,002 (96.2) | **8.1 %** | 1,188 (95.2) | **4.8 %** | 1,116 (95.4) | **4.5 %** |
| Moderate preterm | 65 (3.12) | 0.3 % | 50 (4.0) | 0.2 % | 45 (3.9) | 0.2 % |
| Very preterm | 15 (0.7) | 0.1 % | 10 (0.8) | >0.1 % | 9 (0.8) | >0.1 % |
|  | | | | | | |
